# Treatment of Asthma by Potassium Iodide

**Published:** 1880-03

**Authors:** 


### TREATMENT OF ASTHMA BY POTASSIUM IODIDE.

EVERYONE knows how difficult it is to cure or even benefit the majority of patients who suffer from asthmatic dyspnœa, whether the asthma be chronic or symptomatic; many medicines have been proposed, but they have failed in their purpose, and have consequently fallen out of use. Prof. See, after a great number of investigations, has at last been led to adopt heroic treatment in nearly every case. He administers Iodide of Potassium at the outset of the disease, in a sufficient dose; that is, at least 1.50 grams, increasing it progressively, to 3.4 grams, and limiting the use of the drug on the appearance of symptoms of iodism. It is found that in this way alone is the action of the remedy really effectual.—*Practitioner.*

